# MRI Compatibility: Automatic Brain Shunt Valve Recognition using Feature Engineering and Deep Convolutional Neural Networks

**DOI:** 10.1038/s41598-018-34164-6

**Published:** 2018-10-30

**Authors:** Luca Giancardo, Octavio Arevalo, Andrea Tenreiro, Roy Riascos, Eliana Bonfante

**Affiliations:** 1 Center for Precision Health, School of Biomedical Informatics, University of Texas Health Science Center, Houston, TX USA; 2 McGovern Medical School, University of Texas Health Science Center, Houston, TX USA; 3 Memorial Hermann Hospital, Houston, TX USA

## Abstract

The aim of this study is to evaluate whether we could develop a machine learning method to distinguish models of cerebrospinal fluid shunt valves (CSF-SVs) from their appearance in clinical X-rays. This is an essential component of an automatic MRI safety system based on X-ray imaging. To this end, a retrospective observational study using 416 skull X-rays from unique subjects retrieved from a clinical PACS system was performed. Each image included a CSF-SV representing the most common brands of programmable shunt valves currently used in US which were split into five different classes. We compared four machine learning pipelines: two based on engineered image features (Local Binary Patterns and Histogram of Oriented Gradients) and two based on features learned by a deep convolutional neural network architecture. Performance is evaluated using accuracy, precision, recall and f1-score. Confidence intervals are computed with non-parametric bootstrap procedures. Our best performing method identified the valve type correctly 96% [CI 94–98%] of the time (CI: confidence intervals, precision 0.96, recall 0.96, f1-score 0.96), tested using a stratified cross-validation approach to avoid chances of overfitting. The machine learning pipelines based on deep convolutional neural networks showed significantly better performance than the ones based on engineered image features (mean accuracy 95–96% vs. 56–64%). This study shows the feasibility of automatically distinguishing CSF-SVs using clinical X-rays and deep convolutional neural networks. This finding is the first step towards an automatic MRI safety system for implantable devices which could decrease the number of patients that experience denials or delays of their MRI examinations.

## Introduction

Magnetic Resonance Imaging (MRI) is an essential tool for the evaluation and diagnosis of several medical conditions which uses a powerful magnetic field and radiofrequency stimulation. Implanted medical devices and metallic foreign bodies with ferromagnetic properties interact with the magnetic field and react to radiofrequency stimulation, resulting in undesirable changes in the implantable devices, such as malfunction, displacement, or thermal effects on the tissues surrounding the implant. Prior to an MRI examination, patients need to undergo a screening process to determine if they have devices or foreign bodies that might cause problems, or devices that need to be scanned under specific parameters to assure safety of the patient and integrity of the implant. If patients are able to provide the information, they will fill a screening sheet with the device information. Implant cards or surgical records complement the information provided by the patient. In routine practice, a significant percentage of patients do not have accurate information about medical devices implanted in their bodies. Based on 50 interviews with staff members involved in scheduling and preparing patients for MRI at Memorial Hermann-Texas Medical Center (one of the leading hospitals in the South and Southwest USA), we estimated that 5–10% of patients referred for MRI do not have conclusive information regarding implanted medical devices. As a consequence, radiologists need to infer the device implanted from visually inspecting the X-rays, which leads to delay in the MRI exam and inefficient use of their time. This problem is exacerbated in the emergency room setting, where many patients are unable to provide information regarding implantable devices due to acute medical conditions and a timely MRI can be essential for life saving procedures. A streamlined method of identifying implantable medical devices is not currently available.

In this work, we focus on cerebrospinal fluid shunt valves (CSF-SVs) a type of implanted device important for hydrocephalus treatment. The current standard of care already requires radiologists to visually compare X-rays to manufacturer’s product manuals or other medical publications^1–3^. Correct identification of CFS-SVs is essential for verifying the compatibility with the MRI machine and to monitor for potential setting changes due to the MR magnetic field^4–8^. In fact, exposing patients with some programmable CSF-SVs to MR imaging procedures poses a significant risk of unintentional changes in shunt settings, even if such devices are not MR incompatible^9–12^. Lavinio *et al*.^13^ tested 5 different types of CSF-SV (Codman Hakim, Miethke ProGAV, Medtronic Strata, Sophysa Sophy and Polaris) and found that, with the exception of the Polaris and ProGAV models, all are prone to unintentional reprogramming when exposed to heterogeneous magnetic fields stronger than 40 mT. For this reason, these valves are considered “MR conditional” according to the American Society for Testing and Materials (ASTM)^14^ and require monitoring and readjusting valve setting after the MRI examination.

**Figure 1:**
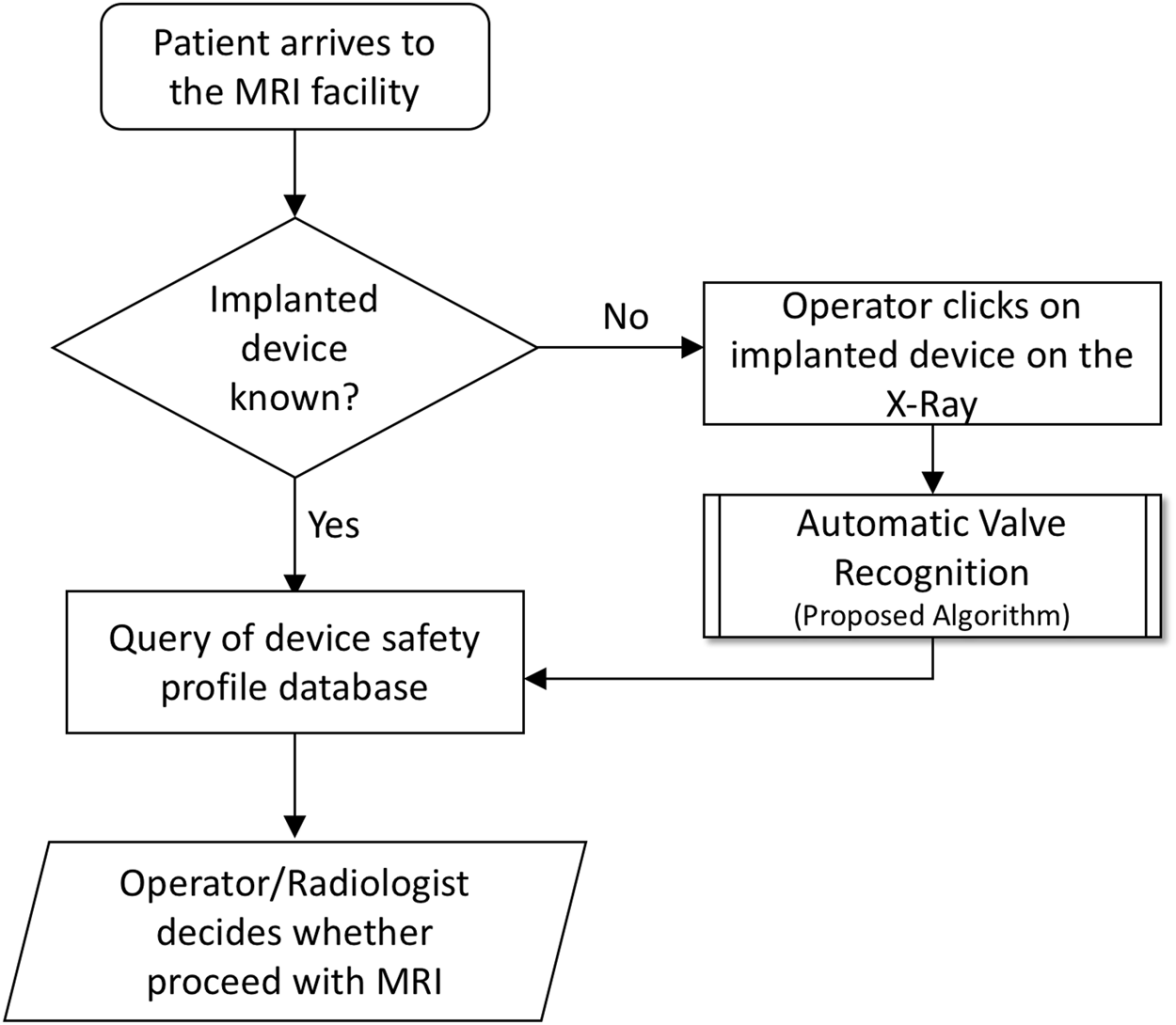
Pipeline of the envisioned system.

In the current standard of care, CSF-SV identification requires coordinated interaction between the MR technologist, the X-ray technologist, the radiologist interpreting the X-ray, and personnel from the referring service. However, this process is suboptimal due to multiple factors, including that non-urgent X-rays have a low level of priority in busy radiology departments, and that the identification process is time consuming and tedious^15^. There are currently 4 different manufacturers of shunt valves in the US market. An image-based system able to automatically identify the main valve model from the X-ray image would allow to immediately display all the safety information required to the radiologist. Based on personal experience from 5 radiologists in our institutions, we estimate that such system has the potential to decrease the X-ray interpretation time by 50–70%, expedite clearance for MRI imaging in emergency conditions, and provide an extra layer of safety for patients and health care providers. We envision an integrated system where a radiologist or X-ray operator could click on the image of the implanted device, a machine learning-based algorithm identifies the type of implanted device, and a database is automatically queried to show the appropriate safety profile (see Fig. 1).

In this study, we compared 4 machine learning pipelines to identify 5 different valves classes on a dataset containing 416 valve X-ray images collected from our clinical PACS system. Two pipelines were based on predefined texture features (Local Binary Patterns and Histogram of Oriented Gradients) and two based on features learned by a deep convolutional neural network architecture. We believe that we are the first group to propose such system and demonstrate its feasibility on clinical data.

## Materials and Methods

### Dataset

The dataset was acquired as a part of a quality improvement project approved by our institution (Quality Improvement Project Registry, no. 2017–017). The UTHealth IRB Office reviewed the study protocol and established that it does not meet the regulatory definition of human subjects research. The methods were carried out in accordance with the relevant guidelines and regulations.

**Figure 2:**
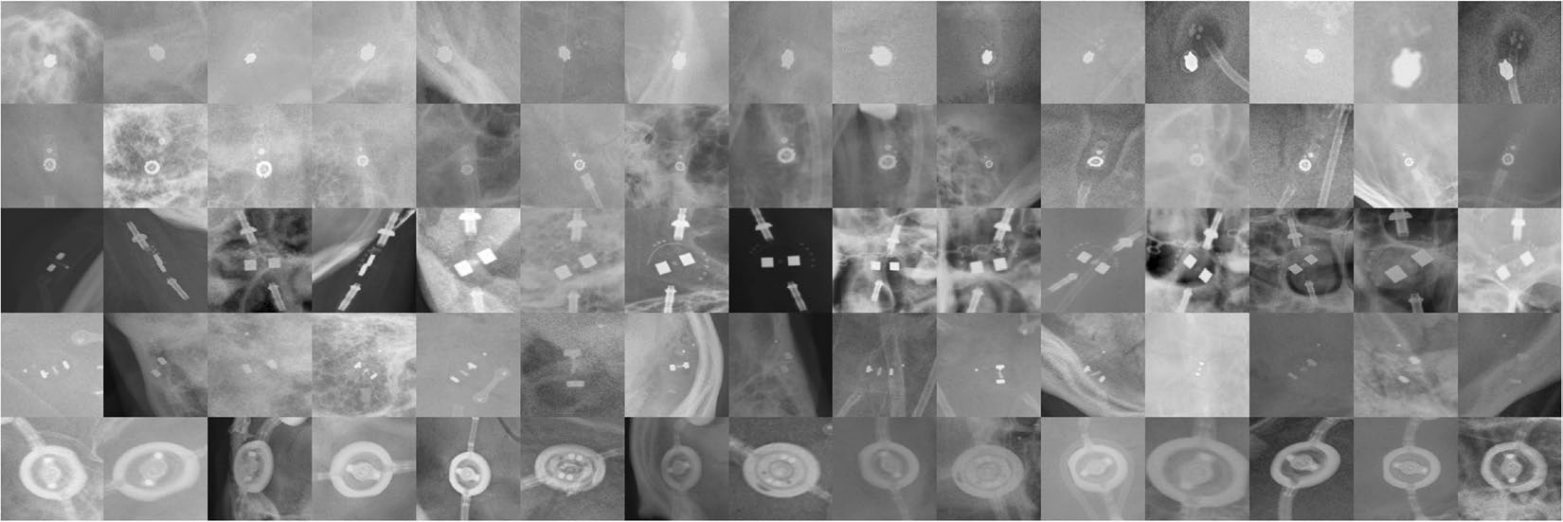
Examples of CSF-SV X-ray valve images in our dataset. Each row shows 15 random samples for each of the 5 classes used. From the top, row 1: *Medtronic Strata II - NSC* (n = 164); row 2: *Codman-Hakim* (n = 106); row 3: *Sophysa Polaris SPV* (n = 82); row 4: *Codman-Certas* (n = 42); row 5: *Miethke ProGAV* (n = 22). In parentheses, the number of samples contained in the dataset. The dataset contains all of the shunt valves brands currently used in US.

A total of 416 skull X-rays that included a CSF-SV image were collected from the institutional PACS. We grouped together different versions of the same CSF-SV models. The specific 5 class grouping is as follows: Codman-Certas® (42), Codman-Hakim® (106), Miethke ProGAV® (22), Sophysa Polaris SPV® (standard/140/400) (82) and Medtronic Strata® (II/NSC) (164). All images were acquired from different subjects and were selected regardless of acquisition perspective, scale or any demographic information. All images were anonymized and an expert radiologist was asked to select an image window of 300 × 300 pixels containing the valve. Such image windows included: valves from different perspectives, scales, brightness, as well as confounding background objects, such as: bone structures, craniotomy hardware or catheters, as shown in Fig. 2. This dataset simulated a system where the X-ray operator or radiologist clicks on an implanted device identified in the X-ray images to retrieve the relevant implanted device safety profile.

### Machine learning pipelines

We developed and tested 4 different machine learning-based pipelines. Each pipeline can be broadly split into an image feature extraction and classification phase. During the image feature extraction phase, a compact numerical representation of the image is generated by encoding the visual information into a fixed size feature vector whose size is significantly less than the number of pixels in the image. These vectors can be created using feature engineering, where measurements are based on a predetermined set of imaging operators or learned, by using representation learning approaches such as the ones implemented by Deep Convolutional Neural Networks (DCNNs)^16,17^. In the classification phase, feature vectors are used as input for a machine learning classifier that will predict the most likely valve type.

### Image feature extraction with feature engineering

The images were pre-processed with histogram normalization which ensures that the dynamic range of the image histogram is between 0 and 1. Then, we implemented two feature extraction pipelines using validated feature engineering approaches, one based on Local Binary Patterns (LBP)^18^ and one based on Histogram of Oriented Gradients (HOG)^19^.

**LBP** features are computed by splitting the image in local windows. Each pixel in the window is compared to its neighbors in order to generate a unique code that represents a characteristic of the texture, such as edges, corners or flat areas. The histogram of these codes is the actual feature vector that will be used as input for the classification phase. More information is available in Ojala *et al*.^18^. In our experiments, we used a multiscale LBP approach using neighbor radius of 6 and 12 pixels, histograms of 30 bins and the LBP codes invariant to image rotations.

**HOG** features are based the image gradients, i.e. the direction/intensity of the image edges. The distribution of these image gradients is computed in local windows that are then concatenated to create the final feature vector. More information is available in Dalal *et al*.^19^. In our experiments we used local windows (or cells) of 20 × 20 pixels.

We leveraged the implementation provided in the scikit-image Python library^20^ for both LBP and HOG.

### Image feature extraction with deep convolutional neural networks (DCNN)

In contrast to feature engineering, representation learning using approaches like DCNN allow for the creation of feature vectors that are not based on a predefined set of rules, but rather learned from the data at hand^16^. However, modern DCNNs often require hundreds of thousands of images for a complete training, also called end-to-end training. Transfer learning is a strategy where a DCNN is first trained on a large dataset containing images unrelated to the problem of interest and then adapted to a smaller dataset. In our experiments, we adopted transfer learning strategies with a modern DCNN architecture as a feature vector generator. Transfer learning had already been used successfully for multiple computational medical imaging problems^21^. We used the Xception network architecture^22^, a DCNN inspired by Inception V3 where convolutional filters are depthwise separable. This network was composed of 126 layers for a total of 22,910,480 trainable parameters (or network weights). A full description of the network is beyond the scope of this article, and we refer the reader to the original publication. In our experiments, we used an Xception network pre-trained on the Imagenet dataset (available at www.image-net.org) which contains over 14 million of hand-annotated natural color images for 1000 classes. The last fully-connected layer of the network was removed and a max pooling layer was added to generate the feature vector. The images were pre-processed using the same histogram normalization as discussed in the previous section, with an additional step to encode monochrome X-ray images into a 3-channel image.

Since the network pre-training was performed on color images, we would normally have to artificially replicate the monochrome intensity value of the X-ray images into 3 channels which the network interprets as RGB. This is the typical approach used by multiple research groups^21^. However, tissues, bones and implanted devices in X-ray images have well defined absorption rates as the X-ray beams traverse matter. Therefore, global thresholds can be effective in differentiating prominent structures from the background. Using this insight, we devised a novel pre-processing strategy allowing us to input additional domain relevant information into the network. In this strategy, we created an image containing a rough estimation of the foreground objects, which was placed into the red and blue channels, while the original X-ray image was left untouched in the green channel. The threshold for foreground objects was estimated using the non-parametric Otsu thresholding approach^23^. In the rest of the manuscript, this step is referred as Enhanced-Xception Network. All DCNNs were implemented using the Keras (www.keras.io) and Tensorflow (www.tensorflow.org) Python libraries.

### Valve classification and statistical analysis

We used the same classification phase for all four feature extraction strategies. The generated feature vector was classified using a linear logistic regression classifier with L2 regularization (the default 1.0 was used as regularization strength). The model was extended to multiclass using a 1 vs all strategy and the coordinate descent optimizer implemented by LIBLINEAR/scikit-learn^24^ was used for training.

We used stratified 10-fold cross-validation to evaluate the performance of the machine learning pipelines. In summary, the dataset was split into 10 chunks (or folds), each fold maintained the same class distribution of the complete dataset. One fold was left out as testing set and the classifier was trained on the remaining 9 folds. This operation was iteratively performed for all folds making sure that the classifier is reset at each iteration. This strategy avoided overfitting and allowed us to robustly estimate the classification performance.

We evaluated the classification performance using *precision* (also known as *positive predictive value)*, *recall* (also known as *sensitivity)* and *F1-score*, i.e. the harmonic mean of precision and recall. All confidence intervals were computed using the non-parametric bootstrap procedure, using 1000 repetitions and reporting the 5th and 95th percentiles. P-values are computed using the two-tailed Mann–Whitney U test to reject the null hypothesis that each valve is indistinguishable from the others in a 1 vs all strategy.

## Results

Table 1 shows the classification performance of the four machine learning pipelines. Deep convolutional networks trained with a transfer learning strategy clearly outperform the two feature engineering methods tested by achieving an accuracy of 95–96% (confidence intervals (CI) [94–97]/[94–98]) vs 56–64% (CI [53–63]/[60–69]). Specifically, the Enhanced-Xception Network performed best for all metrics evaluated (precision: 0.96 CI [0.95–0.98], recall: 0.96 [0.94–0.98]).

**Table 1:** Classification performance of the 4 machine learning pipelines. In brackets the confidence intervals are shown. The accuracy column shows the absolute percentage of images correctly classified.

		Accuracy	Avg. Precision	Avg. Recall	Avg. F1-score
*Feature Engineering Methods*	Local Binary Patterns (LBP)	64% [60–69]	0.64 [0.60–0.69]	0.64 [0.60–0.69]	0.64 [0.60–0.68]
	Histogram of Oriented Gradients (HOG)	56% [53–63]	0.57 [0.53–0.63]	0.56 [0.52–0.61]	0.51 [0.47–0.56]
*Deep Convolutional Neural Networks (Transfer Learning)*	Xception Network	95% [94–97]	0.95 [0.94–0.97]	0.95 [0.94–0.97]	0.95 [0.93–0.97]
	Enhanced-Xception Network	**96% [94–98]**	**0.96 [0.95–0.98]**	**0.96 [0.94–0.98]**	**0.96 [0.94–0.98]**

The precision, recall and f1-score columns represent the performance metrics averaged over the 5 classes. The performance metric for each class is available in Table 2. Deep convolutional networks trained with a transfer learning strategy clearly outperform the two feature engineering methods tested. Specifically, the “Enhanced-Xception Network” is the one showing the best performance overall. (*Precision* is also known as *positive predictive value*; *recall* is also known as *sensitivity*; *F1-score* is the harmonic mean of precision and recall).

**Figure 3:**
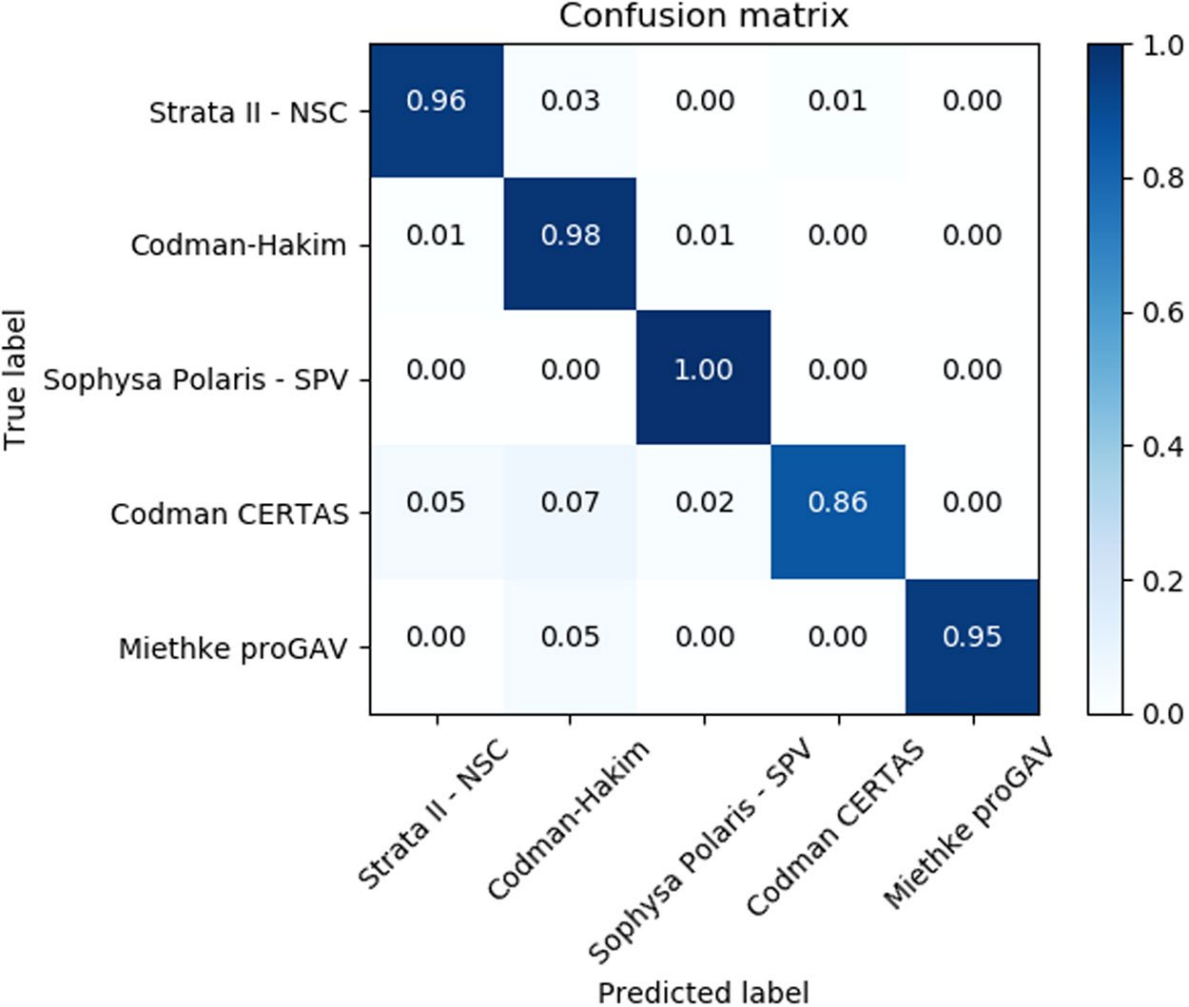
Confusion matrix for the Enhanced-Xception Network. Each cell shows the ratio of images of a given class (true label) classified into the class indicated by the column (predicted label). A perfect classifier will only have 1.00 in the matrix diagonal.

In Table 2, we investigate the performance metric for each valve. All valves were classified with a F1-score equal to or above 0.90, which ranged from 0.99 on the Sophya Polaris SPV class to 0.90 with the Codman-Certas. The F1-score is computed as the harmonic mean of precision and recall. Figure 3 shows the confusion matrix indicating the correct and wrong classification by true valve class and predicted valve class. No obvious misclassification bias was apparent in the matrix.

**Table 2:** Class-level performance metrics using the Enhanced-Xception Network.

	N	Precision	Recall	F1-score	
Strata II - NSC	164	0.98	0.96	0.97	^***^p < 0.001
Codman-Hakim	106	0.92	0.98	0.95	^***^p < 0.001
Sophysa Polaris SPV	82	0.98	1.00	0.99	^***^p < 0.001
Codman-Certas	42	0.95	0.86	0.90	^***^p < 0.001
Miethke proGAV	22	1.00	0.95	0.98	^***^p < 0.001

N = number of samples per class. (*Precision* is also known as *positive predictive value*; *recall* is also known as *sensitivity*; *F1-score* is the harmonic mean of precision and recall). The p-value is computed using the two-tailed Mann–Whitney U test to reject the null hypothesis that each valve is indistinguishable from the others in a 1 vs all framework.

After training, all four machine pipelines could be run in under 1 second per image on a 3.0 Ghz Xeon desktop with a Titan X GPU. Specifically, LBP: ~0.13 seconds/image; HOG: ~0.08 seconds/image; Xception Network ~0.53 seconds/image; Enhanced-Xception Network: ~0.54 seconds/image. The code to replicate our comparative feature analysis is available at https://github.com/lgiancaUTH/shunt-valve-mri-compatibility.

## Discussion

**Figure 4:**
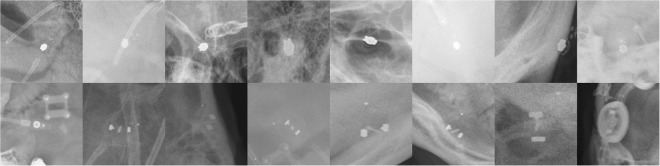
All 16 misclassified samples by the Enhanced-Xception Network. Large foreign object, low contrast or acquisition angles not well represented in the dataset are visible.

In this work, we described a X-rays-based automatic implanted device identification system for MRI safety and devised a pilot study to test the feasibility of the automatic CSF-SV recognition component. We designed four different machine learning pipelines and our results indicate that a deep learning based algorithm (Enhanced-Xception Network) can achieve a very high accuracy (96%) in identifying the valves correctly. We visually inspected the 16 (out of 416 images) that were misclassified (see Fig. 4). In all cases, we noticed large foreign objects, low contrast or acquisition angles not well represented in the dataset. These issues are likely to be solved by increasing the dataset size and including an automatic quality assurance algorithm that would warn the user if the quality of the image is too low for a reliable valve identification. In general, the algorithm was able to classify very challenging samples of valves imaged at skewed angles, scales and location in the skull as shown in Fig. 2.

The best performing algorithm can be run in real time on commodity hardware, thereby making it possible to integrate it on X-ray machines, hospital picture archiving and communication systems (PACS) or software-as-a-service (SaS) cloud services. Additionally, the proposed approach does not require any type of protected health information (PHI), thereby drastically reducing security concerns in the translation of the project to clinical practice.

This study presents some limitations. First, being a retrospective study based on clinical data, we have a different number of samples for each valve type. In order to address the issue, we combined different versions of the same valve type if discrimination between versions was not clinically useful. In our analysis, we could not identify clear classification biases due to class imbalances. Secondly, the automatic image recognition component, while achieving excellent classification performance, did not achieved the performance required for a truly automatic MRI compatibility system. However, in the envisioned workflow shown in Fig. 1, we will always have a human visually comparing the valve models identified by the automated system with the actual X-ray image. Therefore, perfect accuracy would not be strictly required. Additional studies applying our valve identification software in clinical practice are underway.

This technology has the potential to be applied to other medical devices thereby enabling a fast and precise MRI clearance process for busy radiology departments and decrease the number of patients that experience denials or delays of their MRI exams. In fact, the algorithm proposed is not specific to a particular type of implanted device and could be readily adapted by retraining the algorithm on other datasets.
